# Ultrabroadband 1D and 2D NMR Spectroscopy

**DOI:** 10.1002/anie.202515467

**Published:** 2025-11-27

**Authors:** Yannik T. Woordes, Kyryl Kobzar, Sebastian Ehni, Benjamin Görling, Franz Schilling, Angelika Seliwjorstow, Zbigniew L. Pianowski, Peter W. Roesky, Stefan Bräse, Jörg Eppinger, Steffen J. Glaser, Burkhard Luy

**Affiliations:** ^1^ Institute for Biological Interfaces 4 – Magnetic Resonance Karlsruhe Institute of Technology (KIT) Kaiserstr. 12 76131 Karlsruhe Germany; ^2^ Institute of Organic Chemistry Karlsruhe Institute of Technology (KIT) Kaiserstr. 12 76131 Karlsruhe Germany; ^3^ School of Natural Sciences Technische Universität München Lichtenbergstraße 4 85747 Garching Germany; ^4^ KAUST Catalysis Center (KCC) King Abdullah University of Science and Technology (KAUST) Thuwal Saudi Arabia; ^5^ Institute of Biological and Chemical Systems Functional Molecular Systems Karlsruhe Institute of Technology (KIT) Kaiserstr. 12 76131 Karlsruhe Germany; ^6^ Institute of Inorganic Chemistry Karlsruhe Institute of Technology (KIT) Kaiserstr. 12 76131 Karlsruhe Germany; ^7^ Department of Nuclear Medicine Technische Universität München (TUM) Ismaninger Straße 22 81675 München Germany; ^8^ Bruker Biospin GmbH & Co. KG Rudolf‐Plank‐Straße 23 76275 Ettlingen Germany; ^9^ Bruker Switzerland AG Industriestr. 26 Fällanden 8117 Switzerland

**Keywords:** Broadband, Multi‐isotope, NMR spectroscopy, Optimal control, Saturation pulses

## Abstract

The chemical shift range of many NMR‐active isotopes cannot be excited in a single experiment by classical hard pulse high‐resolution spectroscopy or even conventional broadband excitation. Such nuclei can be addressed by specifically optimized saturation pulses or *xy*‐excitation, which are derived from linear frequency sweeps that are further optimized using methods derived from optimal control theory. A multi‐isotope 1D experiment covering 6 MHz as well as homonuclear COSY and heteronuclear HMBC experiments covering more than 100 kHz are demonstrated, which can be adapted to fit any needs for specific isotopes at any spectrometer field. In general, the approach is very useful for 1D and 2D absolute value overview spectra at high magnetic fields and/or wideband and low‐gamma nuclei.

## Graphical abstract

Optimal control‐derived saturation pulses allow 1D and 2D NMR spectroscopy covering spectral ranges in the MHz range. Next to a multitude of nuclei even multinuclear spectra are possible.

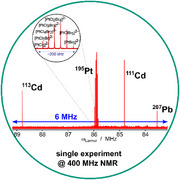

In chemistry, ^1^H and ^13^C NMR spectroscopy certainly dominate everyday laboratory life, but also other NMR‐active nuclei provide highly interesting information and are measured regularly worldwide. Especially the non‐metallic isotopes ^15^N, ^19^F, and ^31^P play an important role in many types of analyses, but also a large number of other nuclei are used particularly in inorganic chemistry. In Figure 1, a selection of such nuclei is compiled with their chemical shift bandwidths Δ*δ* visualized by bars of different lengths, demonstrating the enormous widths that some nuclei comprise. Referenced to a 14.1 T/600 MHz NMR spectrometer, nuclei like ^51^V, ^119^Sn, ^69^Cu as well as ^31^P and organofluorine compounds cover several hundred kHz, and when we consider ^129^Xe, ^195^Pt, ^59^Co, and the full range of ^19^F chemical shifts, even chemical shift ranges in the MHz range apply.^[^ ^1^, ^2^ ^]^ The values obviously scale with the magnetic field and readily installed high‐field spectrometers at 28.2 T/1200 MHz, as well as currently discussed 35.3 T/1500 MHz spectrometers, will have even larger Δ*δ* ranges with corresponding scaling factors of 2.0 and 2.5, respectively. In modern 1D and 2D hard pulse Fourier transform NMR spectroscopy, on the other hand, the bandwidth Δ*Ω* that can be excited without severe compromise in sensitivity is approximately given by the so‐called Rabi frequency or rf‐amplitude *ν*_rf_ of a particular experimental setup. In high‐resolution NMR spectroscopy, essentially independent of the magnetic field strength, this maximum rf‐amplitude typically ranges from 31 kHz (corresponding to an 8 µs 90° hard pulse) for nuclei with high gyromagnetic ratios *γ* and probeheads with the detected nucleus on the inner coil to 7.1 kHz (corresponding to a 35 µs 90° hard pulse) for low *γ* nuclei on an outer coil. Correspondingly, the number of experiments required to record the full range of a nucleus of interest is approximately given by the ratio Δ*δ*/Δ*Ω* ≈ Δ*δ*/*ν*_rf_. As such, even ^15^N as a low *γ* heteronucleus with an overall bandwidth below 100 kHz at 14.1 T poses a severe problem with a ratio of Δ*δ*/*ν*_rf_ = 12.6, i.e., 13 experiments are to be acquired to cover the entire chemical shift range, while at least 250 experiments would be necessary to cover, e.g., the full ^19^F or ^59^Co range. Although this large number can usually be reduced significantly by prior knowledge of the compound classes to be expected, it would still be good to be able to cover the entire range Δ*δ* in a single experiment, as one can never exclude that unexpected side reactions take place. We therefore put our efforts into developing shaped pulses with a bandwidth Δ*Ω* ≥ Δ*δ*, which can cover any of the desired ranges with a standard spectrometer setup with readily accessible pulse lengths and rf energies.

**Figure 1:**
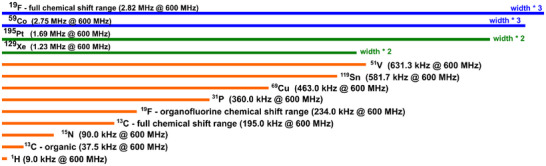
Chemical shift ranges of various nuclei at a magnetic field strength of 14.1 T/600 MHz. Chemical shift ranges are visualized by colored horizontal bars. While conventional chemical shift ranges of ^1^H and ^13^C can be excited with sufficiently short hard pulses, all other presented nuclei require the acquisition of several hard pulse experiments with shifting irradiation frequencies to detect all potential signals. Chemical shift ranges shown with green bars are twice, and with blue bars three times as wide as shown.

The most essential pulse in NMR spectroscopy is an excitation pulse, typically a 90° pulse, for which three different types of shaped pulses can be applied. The first type is a universal rotation^[^ ^3^ ^]^ or class‐A^[^ ^4^ ^]^ pulse, which transforms all magnetization components as if it would be a hard 90° pulse on‐resonant. Such pulse shapes are very demanding and although systematic searches have been performed,^[^ ^3^ ^]^ the largest Δ*Ω*/*ν*_rf_ ratio reported so far is 6,^[^ ^5^ ^]^ leaving this class of pulses inappropriate for really large chemical shift ranges. The second type is a point‐to‐point excitation or class‐B2^[^ ^4^ ^]^ pulse, which only excites a single component—usually polarization along *z*—onto a specific axis in the *x*,*y*‐plane, for example, the *x*‐axis (Figure 2a). Such pulse shapes have been systematically studied up to Δ*Ω*/*ν*_rf_ = 6,^[^ ^6^, ^7^ ^]^ but singular pulse shapes have been reported with the ABSTRUSE,^[^ ^8^ ^]^ CHORUS,^[^ ^9^ ^]^ and corrected CHORUS^[^ ^10^ ^]^ composite adiabatic pulses, reaching even Δ*Ω*/*ν*_rf,max_ ≈ 30. But pulse shapes get very long, and the corresponding corrected CHORUS pulse has a normalized duration *t*_p_∙*ν*_rf,max_ = 71.48. Larger bandwidth pulses can in principle be calculated, but their pulse length would be intolerable in most experiments. This leaves the third type of pulses, saturation or class‐B3^[^ ^4^ ^]^ pulses, which transfer *z* polarization into the *x*,*y*‐plane without defining a particular phase, i.e., depending on the chemical shift offset spins will be excited with a different phase (Figure 2b). We may refer to them also as *xy*‐excitation pulses. As a consequence, resulting spectra should either be processed using their absolute value or by using a specific, computer‐simulated phase profile. This type of pulse shape has been used for excitation in ultrafast experiments^[^ ^11^, ^12^ ^]^ and EPR spectroscopy.^[^ ^13^, ^14^ ^]^ Again, pulse shapes up to approximately Δ*Ω*/*ν*_rf_ = 30 have been reported.^[^ ^13^ ^]^ All of these saturation pulses involve adiabatic pulse shapes, either WURST,^[^ ^15^, ^16^ ^]^ CHIRP,^[^ ^17^ ^]^ or hyperbolic secant^[^ ^18^, ^19^ ^]^ pulses. Particularly linear frequency‐swept CHIRP pulses show well‐acceptable performance at very short pulse durations. But their excitation profile shows an inherent, offset‐dependent modulation of the excited transverse magnetization intensities,^[^ ^13^ ^]^ so we decided to use randomized pulses as well as linear frequency sweeps as the starting point for optimal control theory (OCT)‐based saturation pulse optimizations.

**Figure 2:**
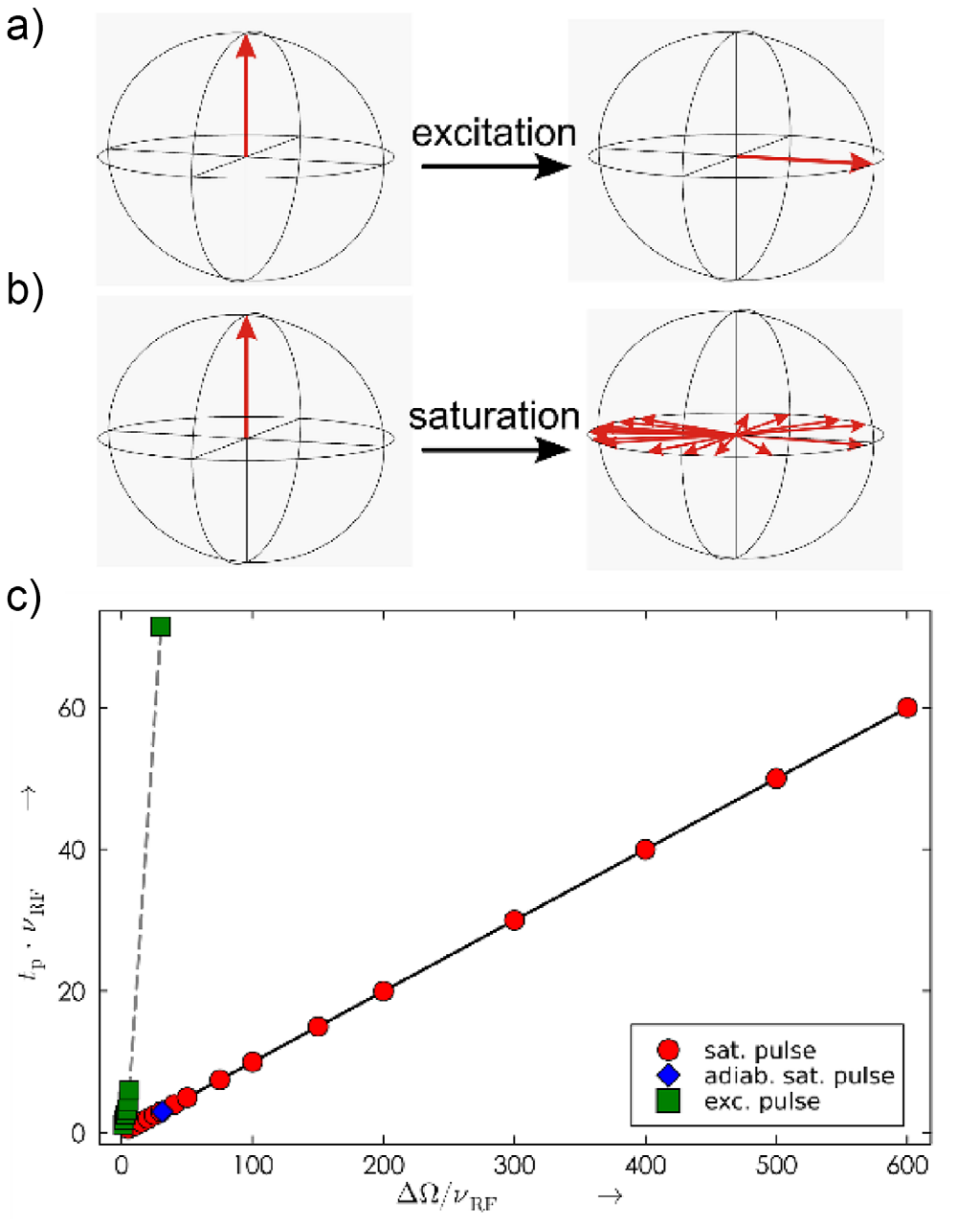
Compilation of previously reported and newly derived broadband pulses to excite large chemical shift ranges. While excitation pulses rotate initial *z*‐polarization to a defined axis in the *x*,*y*‐plane a), saturation (or *xy*‐excitation) pulses are less restrictive, allowing the initial polarization to be spread with an offset‐dependent phase in the *x*,*y*‐plane b). Systematically derived time‐optimal pure phase BEBOP excitation pulses, (^[^ ^6^, ^7^ ^]^ green boxes), a corrected bandwidth‐optimized adiabatic CHORUS with Δ*Ω*/*ν*_rf_ ≈ 30, (^[^ ^10^ ^]^ green box), a CHIRP‐type adiabatic saturation pulse with Δ*Ω*/*ν*_rf_ ≈ 30, (^[^ ^13^ ^]^ blue diamond), and the OCT‐derived xyBEBOP saturation pulse shapes from this publication (red circles) c). Pulses are characterized according to their rf‐amplitude‐normalized pulse length *t*_p_·*ν*_rf_ = *t*_p_/(4 *t*_90°_) and bandwidth Δ*Ω*/*ν*_rf_.= Δ*Ω*·4 *t*_90°_, where *t*_90°_ represents the 90° pulse length for the maximum rf‐amplitude of the shape.

As has been demonstrated in many examples,^[^ ^20^, ^21^, ^22^, ^23^, ^24^, ^25^, ^26^, ^27^, ^28^, ^29^, ^30^, ^31^, ^32^, ^33^, ^34^ ^]^ OCT‐derived algorithms allow the optimization of pulse shapes without any shape limitation. Singular so‐called xyBEBOP (derived from *xy*‐excitation and the previous acronym Broadband Excitation By Optimized Pulses (BEBOP) for conventional optimal control excitation pulses^[^ ^23^ ^]^) saturation pulses have already been optimized using OCT‐algorithms, demonstrating their high potential.^[^ ^35^, ^36^, ^37^, ^38^ ^]^ A systematic study of saturation pulses of intermediate bandwidths revealed that best‐performing amplitude‐restricted saturation pulses show quasi‐adiabatic pulse shapes with a roughly linear frequency sweep, which, however, is highly modulated (manuscript in preparation, see also zoomed regions of the phase profiles for all *xy*BEBOP pulses of this article in the Supporting Information). We therefore focused on constant amplitude linear frequency sweeps as starting pulses for pulse optimizations, where the sweep rates were varied along the ranges provided in Refs. [12, 13]. Resulting optimized pulse shapes show exceptional performance over the entire optimization bandwidth, which is also reproduced experimentally (Figure 3).

As a first application, we looked into ^195^Pt spectroscopy, where we tried to reproduce data for K_2_PtBr_6_^[^ ^1^ ^]^ and all variants of K_2_PtBr*_n_* Cl_(6‐_ *_n_*_)_ down to K_2_PtCl_6_ by adding HCl to the neat starting compound. The spectrum comprises almost 2000 ppm or a bit less than 200 kHz on a 400 MHz spectrometer, representing slightly less than one‐eighth of the entire ^195^Pt chemical shift range. However, we soon realized that we can go far beyond this and prepared a sample with Cd‐acetate and Pb‐acetate with altogether four different spin ½ isotopes: ^113^Cd, ^195^Pt, ^111^Cd, and ^207^Pb. The multi‐isotope spectrum of the sample requires a spectral width of about 5.5 MHz, while our standard 400 MHz BBO‐probehead allows a 90° hard pulse of approximately 14 µs. We therefore chose a pulse shape with a Δ*Ω*/*ν*_rf_ ratio of 400 at an rf‐amplitude *ν*_rf_ = 15 kHz, resulting in a pulse duration *t*_p_ of 2666.6 µs. The corresponding pulse shape with its theoretical and experimental offset profile is shown in Figure 3. Indeed, the 6 MHz excitation bandwidth is sufficient to excite all four isotopes in a single 1D experiment, which is shown in Figure 4 together with a zoom of the ^195^Pt sub‐spectrum. As tuning and matching on our standard spectrometer and probehead are not able to provide a uniform rf‐amplitude over the entire 6 MHz bandwidth, signal intensities of the ^113^Cd and ^207^Pb signals are reduced to approximately 35% relative to the on‐resonant excitation while the effect on ^195^Pt and ^111^Cd signals is negligible (see the Supporting Information for a detailed analysis of the ^207^ Pb signal). The effect is similar to offset‐dependent hard pulse excitation and may be overcome by probehead and spectrometer‐specific optimized pulse shapes or hardware design, which, however, is beyond the demonstration purpose we aim for in this article. It should rather be noted that the 6 MHz excitation bandwidth is equally sufficient to fully excite the chemical shift range of any of the nuclei summarized in Figure 1 at any currently available magnetic field strength, including the very recently manufactured 1300 MHz high‐resolution NMR spectrometer.^[^ ^39^ ^]^ With the Δ*Ω*/*ν*_rf_ = 600 pulse from Figure 2, finally, this bandwidth can be achieved with an rf‐amplitude of only 10 kHz.

**Figure 3:**
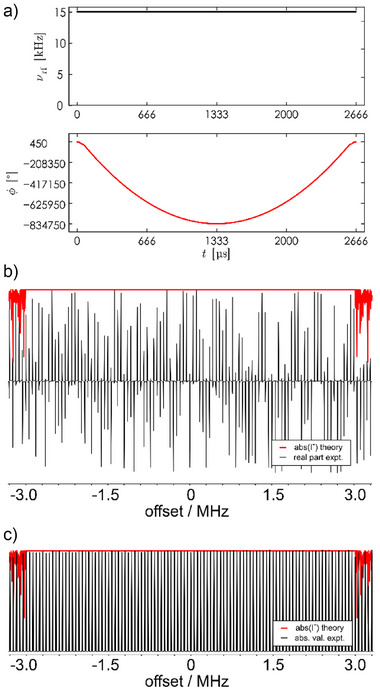
Characterization of the Δ*Ω*/*ν*_RF_ = 400 *xy*BEBOP saturation pulse of Figure 1. Rf‐amplitude *ν*_rf_ and phase *ϕ* (a) as well as offset profiles (b) and (c) are given. Theoretical profiles for the transfer of *z*‐magnetization into the *x*,*y*‐plane are drawn in red on top of experimental data. In (b), for the experimental profile 111 1D experiments with increasing offsets of the shaped pulse have been acquired and processed phase sensitively, indicating the strongly offset‐dependent spectral phase of the saturation pulse excitation. With the very same experiments processed using absolute values, the offset profile shows an almost constant excitation over the entire 6 MHz bandwidth (c). Experiments were acquired using the residual HDO signal of a 600 µL‐doped D_2_O sample.

**Figure 4:**
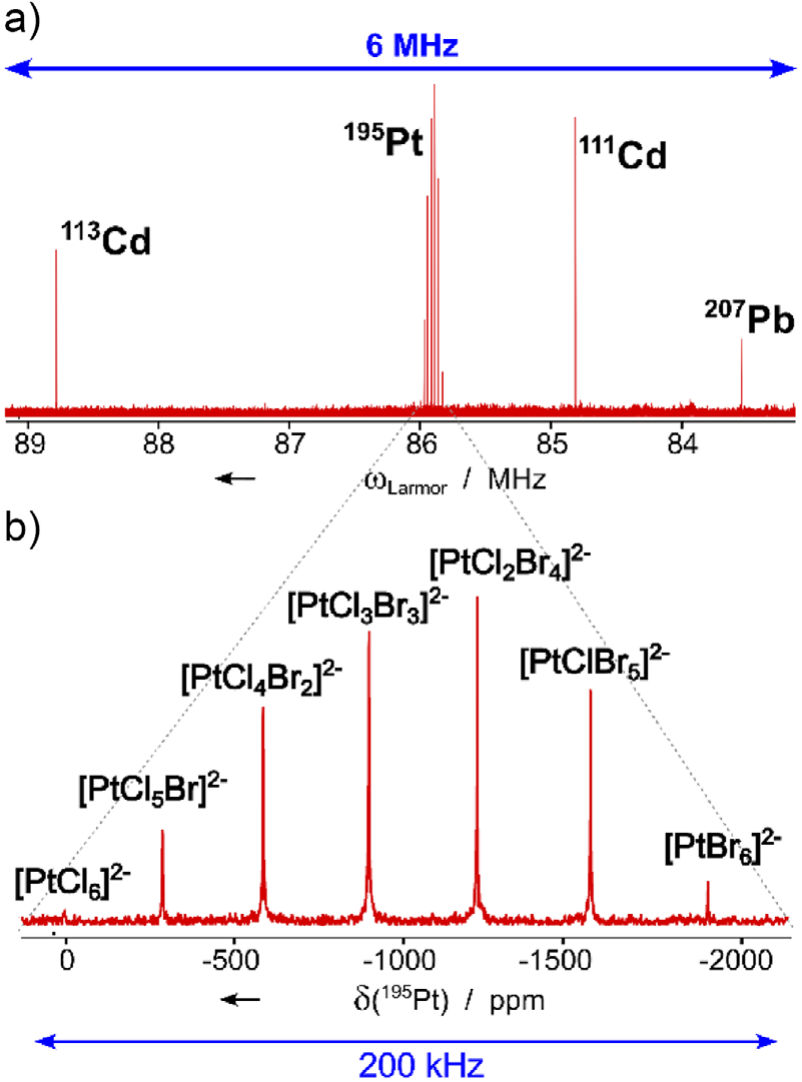
Multi‐isotope spectrum comprising ^113^Cd, ^195^Pt, ^111^Cd, and ^207^Pb acquired in 12 h using the sample described in detail in the main text. The 6 MHz bandwidth spectrum was acquired on a 9.4 T/400 MHz spectrometer with a standard probehead optimized for the detection of heteronuclei using the pulse shape characterized in Figure 3 for uniform broadband excitation (a). The ^195^Pt spectrum reveals the well‐known distribution of K_2_PtBr*_n_* Cl_(6‐_ *_n_*_)_ components (b).^[^ ^1^ ^]^

While the multi‐isotope spectrum demonstrates the bandwidth capabilities of saturation pulses, they can also be used to enhance the bandwidth in standard 2D experiments. In a COSY experiment, for example, the excited magnetization is evolving during the *t*_1_ evolution period and irrespective of its initial phase, the resulting antiphase terms will be rotated in such a way that they are transferred to give a signal with the frequency of a directly coupled nucleus. This can now be achieved with any bandwidth up to 6 MHz. The example for a ^19^F,^19^F‐COSY is given in Figure 5c using a 200 µs long Δ*Ω*/*ν*_rf_ = 20 pulse, covering 200 kHz spectral width. For the compound 2,2,3,4,4,4‐hexafluoro‐1‐butanol cross peaks on a 600 MHz spectrometer span a frequency range of approximately 80 kHz. While the spectrum with the saturation pulses shows intense correlations, the corresponding spectrum using the shortest possible 90° ^19^F hard pulse with 24.25 µs is essentially void of the desired signals (Figure 5b). Please note that the shown bandwidth of 100 kHz cannot be covered by any conventional pulse shape of acceptable duration (≤1 ms) at the available rf‐amplitude. The same experiment with the 200 µs *xy*BEBOP‐shaped pulses would also cover the same ppm‐bandwidth of Figure 5c on a 1.2 GHz spectrometer.

**Figure 5:**
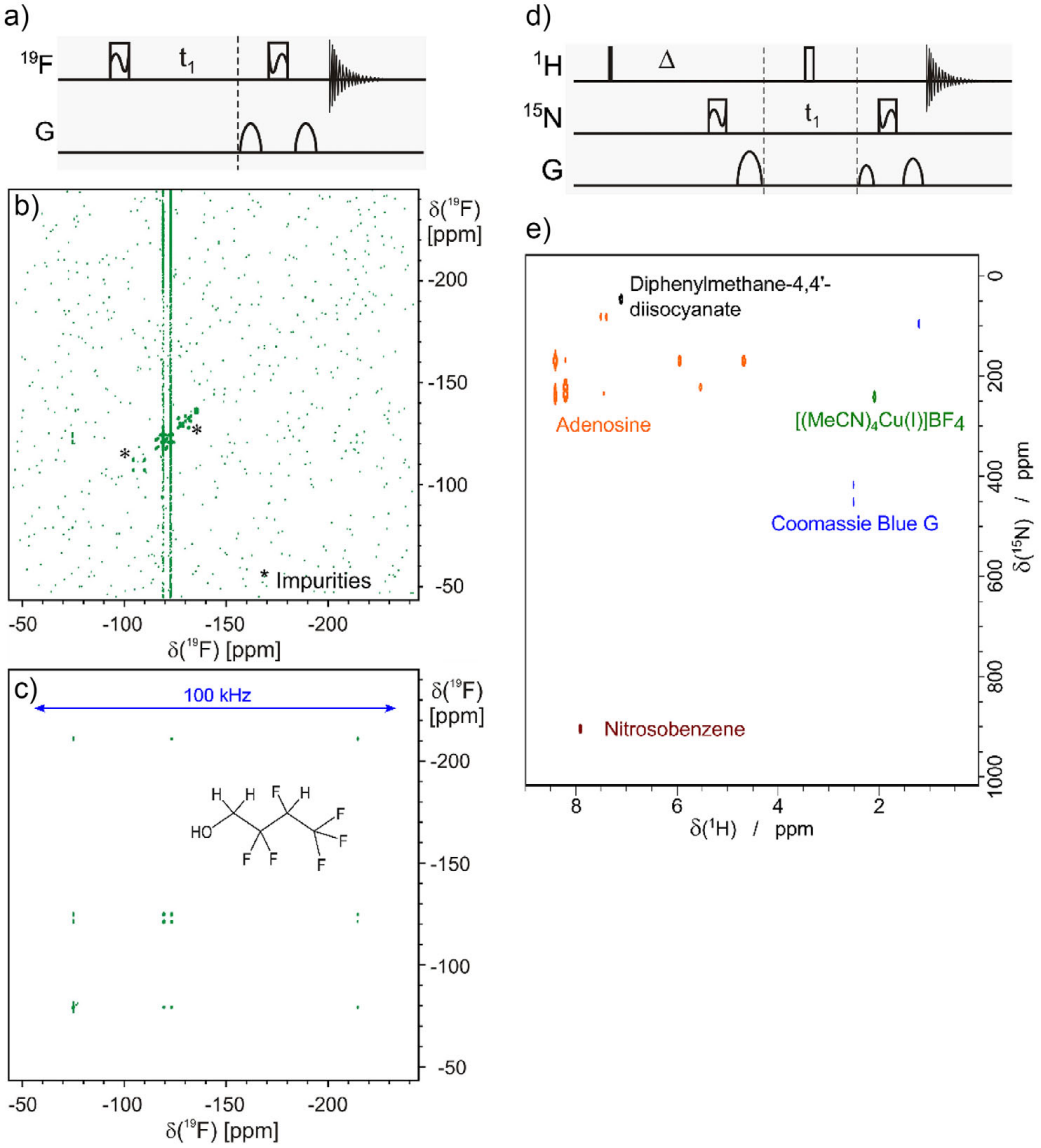
Example 2D experiments acquired using *xy*BEBOP saturation pulses. a) ^19^F, ^19^F‐COSY pulse sequence scheme, in which *xy*BEBOP pulse shapes are indicated by boxes filled with a wavy line. b) The pulse sequence applied to 2,2,3,4,4,4‐hexafluoro‐1‐butanol on a 14.1 T/600 MHz spectrometer using the 24.25 µs hard pulse available on the HCNF‐QXI probehead and c) same experiment using 200 µs Δ*Ω*/*ν*_rf_ = 20 *xy*BEBOP pulses. While the hard pulse version misses out most correlations down to the noise level, the *xy*BEBOP version provides high‐quality spectra. It should be noted that signals from impurities are also present in the *xy*BEBOP spectra, but all signals of interest are visible already at higher contour levels. d) ^1^H, ^15^N‐HMBC pulse sequence scheme with xyBEBOP pulses on nitrogen. e) ^1^H, ^15^N‐HMBC spectra were measured for five compounds as indicated in the spectral overlay, covering a chemical shift range of approximately 900 ppm. Spectra were acquired with identical parameters on a 20 T/850 MHz spectrometer involving two 697.7 µs Δ*Ω*/*ν*_rf_ = 30 *xy*BEBOP pulses with a bandwidth of 129 kHz/1500 ppm.

Also heteronuclear 2D experiments can be realized with saturation pulses. A particular experiment of interest is a ^1^H, ^15^N‐HMBC, which is typically acquired on inverse‐type probeheads with nitrogen on an outer coil. The low‐γ nucleus ^15^N in this case has very long 90° hard pulses of typically 35 µs or longer. The large chemical shift range of approximately 1500 ppm is out of reach, unless broadband excitation is used. Using a 697.7 µs *xy*BEBOP pulse shape with Δ*Ω*/*ν*_rf_ = 30 at an rf‐amplitude *ν*_rf_ = 4.3 kHz, a bandwidth of 129 kHz is reached, covering the entire frequency range on spectrometer fields up to 20 T, i.e., 850 MHz ^1^H Larmor frequency. To demonstrate the capabilities of such an experiment, we acquired ^1^H, ^15^N‐HMBC spectra on diphenylmethane‐4,4′‐diisocyanate, adenosine, [(MeCN)_4_Cu(I)]BF_4_, coomassie brilliant blue G, and nitrosobenzene on an 850 MHz spectrometer with identical setup and overlaid them to a single spectrum shown in Figure 5e. Clearly, the 900 ppm bandwidth of the compounds is easily covered.

In summary, we present a number of *xy*BEBOP saturation pulses that generally enable the coverage of chemical shift ranges of all isotopes at all currently available static magnetic field strengths, as long as homogeneous *T*_2_ relaxation times of acquired signals are longer than the pulse length *t*_p_. Most diamagnetic and particular paramagnetic samples^[^ ^40^ ^]^ will benefit from the pulse shapes, where, however, the *T*_2_ restriction will lead to reduced signal intensities and lineshape distortions to heavily broadened signals. Next to 1D experiments also homo‐ and heteronuclear 2D experiments with 90° pulses on the broadband isotope can be performed. Even the acquisition of multi‐isotope spectra is possible, although analog‐to‐digital converters and filtering in current spectrometers limit the bandwidth to several MHz. Resulting spectra should best be processed using absolute values as done here, but also phase‐sensitive processing seems amenable, as phase‐offset profiles are easily calculated. Future developments using cooperative saturation‐type s^2^‐COOP/RAM‐COOP pulses^[^ ^41^, ^42^ ^]^ can be imagined, which would simplify the processing for absorptive‐phase spectra.

With the availability of the presented pulse shapes, chemists have an important tool in hand to record entire NMR spectra of nuclei routinely in a single experiment, which will have an impact on measurement time and will enable the performance of experiments that so far would not have been feasible.

## Supporting Information

Materials and methods as well as theoretical offset profiles of all pulse shapes with Δ*Ω*/*ν*_rf_ ratios ranging from 5 to 600 are provided in the Supporting Information. In addition, all original datasets, pulse shapes, and pulse programs (Bruker) are provided under the DOI: 10.35097/0h60k266a6z74210.

## Conflict of Interests

The authors declare no conflict of interest.

## Supporting information



Supporting Information

## Data Availability

The data that support the findings of this study are openly available in [KITOpen] at [https://doi.org/10.35097/0h60k266a6z74210], reference number [1000183140].
