# Supplementary material for: Ultrabroadband 1D and 2D NMR Spectroscopy
Source: Angew Chem Int Ed Engl. 2025 Nov 27;65(2):e15467. doi: 10.1002/anie.202515467 (PMC12790362; doi:10.1002/anie.202515467)
Supplement: Supplementary file 1 — Supporting Information [file ANIE-65-e15467-s001.docx]

Supporting Information

for

Ultrabroadband 1D and 2D NMR Spectroscopy

Yannik T. Woordes,^[a]^ Kyryl Kobzar,^[h]^ Sebastian Ehni,^[i]^ Benjamin Görling,^[h]^ Franz Schilling,^[g]^ Angelika Seliwjorstow,^[b]^ Zbigniew L. Pianowski,^[b,e]^ Peter W. Roesky,^[f]^ Stefan Bräse,^[b,e]^ Jörg Eppinger,^[d]^ Steffen J. Glaser,^[c]^ and Burkhard Luy*^[a],[b]^

Dedicated to Richard R. Ernst (1933-2021)

[a] Y. T. Woordes, Prof. Dr. B. Luy*
Institute for Biological Interfaces 4 – Magnetic Resonance
Karlsruhe Institute of Technology (KIT)
Hermann-von-Helmholtz-Platz 1
76344 Eggenstein-Leopoldshafen
Germany
E-mail: Burkhard.Luy@kit.edu

[b] PD Dr. Z. Pianowski, Prof. Dr. S. Bräse, Prof. Dr. B. Luy
Institute of Organic Chemistry
Karlsruhe Institute of Technology (KIT)
Fritz-Haber-Weg 6
76131 Karlsruhe
Germany

[c] Prof. S. J. Glaser
Institute of Organic Chemistry
Technische Universität München
Lichtenbergstraße 4
85747 Garching
Germany

[d] Prof. J. Eppinger
KAUST Catalysis Center (KCC)
King Abdullah University of Science and Technology (KAUST)
Thuwal
Saudi Arabia

[e] PD Dr. Z.L. Pianowski, Prof. Dr. S. Bräse,
Institute of Biological and Chemical Systems
Functional Molecular Systems
Karlsruhe Institute of Technology (KIT)
Hermann-von-Helmholtz-Platz 1
76344 Eggenstein-Leopoldshafen
Germany

[f] Prof. Dr. P. W. Roesky
Institute of Inorganic Chemistry
Karlsruhe Institute of Technology (KIT)
Engesserstr. 15
76131 Karlsruhe
Germany

[g] Prof. Dr. F. Schilling
Department of Nuclear Medicine
Technische Universität München (TUM)
Ismaninger Straße 22
81675 München
Germany

[h] Dr. K. Kobzar and Dr. B. Görling
Bruker Biospin GmbH & Co. KG
Rudolf-Plank-Straße 23
76275 Ettlingen
Germany

[i] Dr. S. Ehni
Bruker Switzerland AG
Industriestr.26
8117 Fällanden
Switzerland

**Experimental**

**^19^F,^19^F-COSY:** An approximately 100 mM sample of 2,2,3,4,4,4-fluoro-1-butanol in CDCl_3_ was measured on a 600 MHz Bruker Avance II spectrometer equipped with a ^1^H,^13^C,^15^N,^19^F-QXI room temperature probehead. The probehead provides a ^19^F hard pulse of 24.25 µs. A standard COSY experiment using coherence selection via gradients has been applied with hard pulses (Bruker pulse sequence cosygpqf) and with rectangular hard pulses replaced by a 190 µs long xyBEBOP pulse (modified pulse sequence buluXYcosygpqf) optimized to cover a bandwidth of 200 kHz (355 ppm) at an rf-amplitude of 10 kHz (corresponding to a 25 µs 90° hard pulse). 1024 times 16384 points were acquired in the indirect and direct dimension with an overall spectral width of 113636 Hz/201.2 ppm in both dimensions. With four dummy scans and 1 scan per increment the experiments were acquired in 19 minutes each. An adapted sine-function was used for apodization in both dimensions and standard absolute value (magnitude calculation mc) processing was applied. While all expected correlations are easily visible in the xyBEBOP COSY spectrum with good intensities (Fig. 5 C in the main text), the hard pulse sequence shows only very few signals of the desired compound. As a consequence, contour levels in Fig. 5 B in the main text are provided at the noise level, where clearly some minor impurity diagonal signals are visible, but desired cross peaks for 2,2,3,4,4,4-fluoro-1-butanol cannot be seen as the approximately 80 kHz spanned by the compound are too wide to be covered by the hard pulse.

**^1^H,^15^N-HMBC:** Altogether five 100-200 mM samples were prepared using the compounds diphenylmethane-4,4’-diisocyanate, adenosine, [(MeCN)_4_ Cu(I)]BF_4_, Coomassie Brilliant Blue G, and nitrosobenzene and 2D-HMBC experiments using hard pulses (Bruker pulse sequence hmbcgpndqf) as well as xyBEBOP shaped pulses (pulse sequence bulu_hmbcgpndqf_sp) on the nitrogen channel were recorded. Spectra were acquired on a Bruker Ascend 850 MHz Avance III HD spectrometer equipped with a ^1^H,^13^C,^15^N-TCI cryoprobe using a ^1^H spectral width of 13.1 ppm (2048 points) and a ^15^N spectral width of 1500 ppm (128 points). The hard 90° pulses were 7.8 µs for protons and 32 µs for nitrogen. The xyBEBOP pulse shape used had a bandwidth-to-rf-amplitude-ratio of 30 and was applied with ν_rf_ = 4300 Hz/*t*_90°_ = 71.5 µs at a shape duration of 697.7 µs to cover 129 kHz bandwidth. All spectra were acquired with 16 dummy scans and 8 scans per increment, leading to a duration of 38 minutes per 2D experiment. Spectra were processed to 2048 times 1024 points using a quadratic sine function for apodization. The spectral widths were chosen to cover the full range of ^15^N of -400 to 1100 ppm using the Bruker internal referencing system.

**Multinuclear 1D:** Potassium hexabromoplatinate(IV), lead(II)acetate trihydrate and cadmium(II)acetate dihydrate were ordered from Sigma Aldrich and used without further purification. Lead(II)acetate trihydrate (201.2 mg, 530.4 mmol) was dissolved in 500 μL deuterated water to a cloudy mixture. The solution was sonicated for 10 minutes until a clear solution was obtained. Potassium hexabromoplatinate(IV) (101.0 mg, 134.2 mmol) was added to 700 μL of deuterated water to a suspension of dark red and light red powder in a light red solution. The addition of a few drops of 37% HCl increased the solubility to about 1/3 of the platinum salt. The suspension was filtered through a syringe and cadmium(II)acetate (30.9 mg, 115.9 mmol) was added. The addition of the cadmium salt was followed by the precipitation of small amounts of solid from the solution. After previous trials to combine all compounds in a single sample failed because of precipitation of at least one of the components, we used the sample with potassium hexabromoplatinate(IV) and cadmium(II)acetate dihydrate in a conventional NMR-tube, to which we added a capillary with the Lead(II)acetate trihydrate sample in the center of the active volume. A 1D experiment was acquired using an xyBEBOP pulse shape with a bandwidth-to-rf-amplitude-ratio of 400, resulting in a pulse shape of 2.666 ms duration at ν_rf_ = 15000 Hz/*t*_90°_ = 16.6 µs, covering a bandwidth of 6 MHz. The 1D experiment of Fig. 4 was acquired on a standard 400 MHz Bruker Avance Neo spectrometer equipped with a room temperature BBO-probehead. 5000000 points per FID were acquired in 51200 scans overnight (15 hours) in 5 experiments that were added before processing. Due to analog-digital-converter restrictions, the spectral width was set to 7.5 MHz, leading to a digital resolution of 3 Hz per spectral point. For a good presentation line-width, a linebroading of 100 Hz was added by an exponential apodization function. The irradiation frequency was set to the center of the expected Larmor frequencies of the four isotopes ranging from ^113^Cd, ^195^Pt, ^111^Cd to ^207^Pb, i.e. at 86.2 MHz. It should be noted that the tuning and matching of the LCR resonator of the probe cannot cover the entire 6 MHz range. The signals of ^113^Cd and ^207^Pb at the edges of the spectral width are therefore slightly attenuated.

**Pulse Optimizations:** xyBEBOP saturation pulses were optimized with a implementation of the GRAPE algorithm with exact gradients using quaternions and analytical solutions for both cost functions and gradients. The self-written code uses a standard Julia Optimizer implementation of L-BFGS. All shapes have constant amplitude and only pulse phases were optimized, leading to overall optimization times of 2 seconds for the ΔΩ/ν_rf_ = 5 pulse shape to approximately 10 days for the longest pulses. For the pulse shapes with ΔΩ/ν_rf_ = 500 and 600 the optimization runs were not yet fully converged when we stopped the optimizations. Still, corresponding pulse shapes show exceptional performance.

**Pulse Shapes and Offset Profiles**

Pulse shapes and theoretical profiles for the different bandwidth-to-rf ratios.

**ΔΩ/ν_rf_ = 5**

**
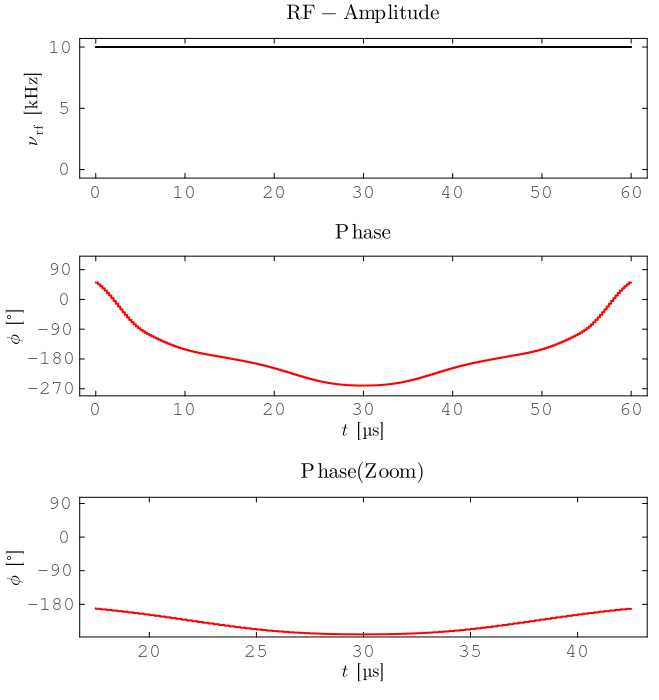

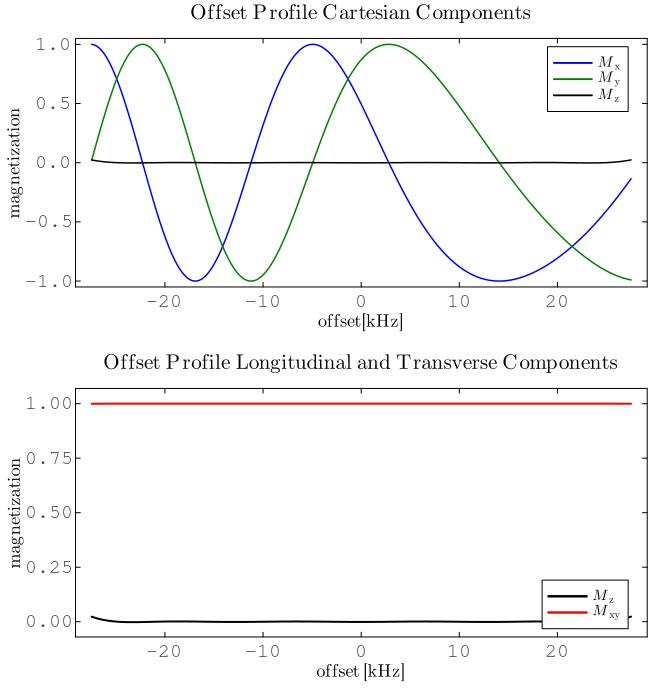
**

**ΔΩ/ν_rf_ = 10**

**
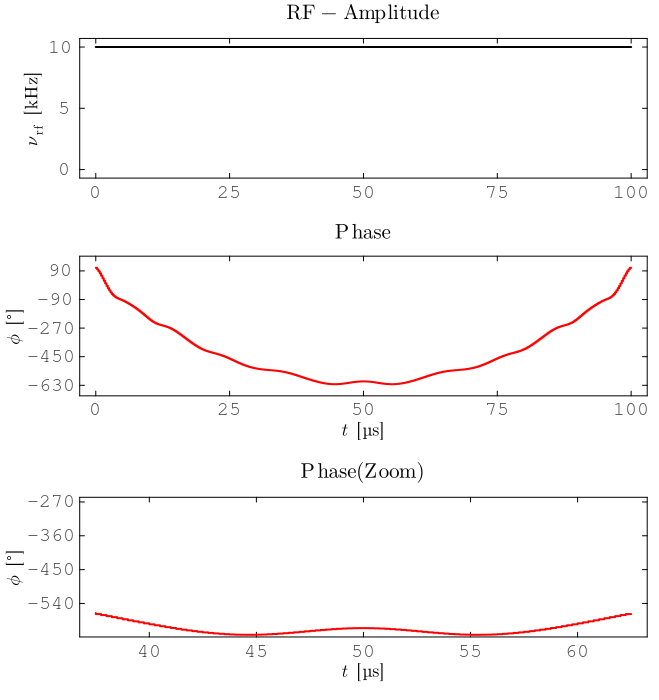

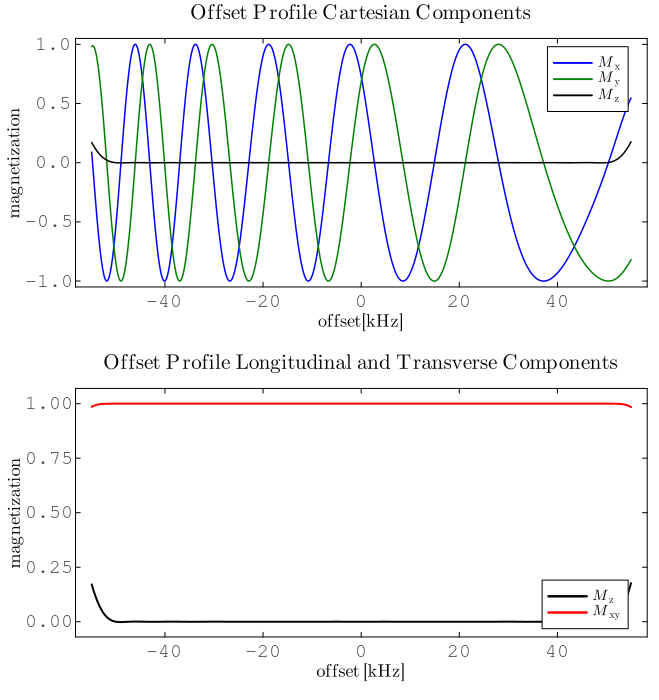
**

**ΔΩ/ν_rf_ = 15**

**
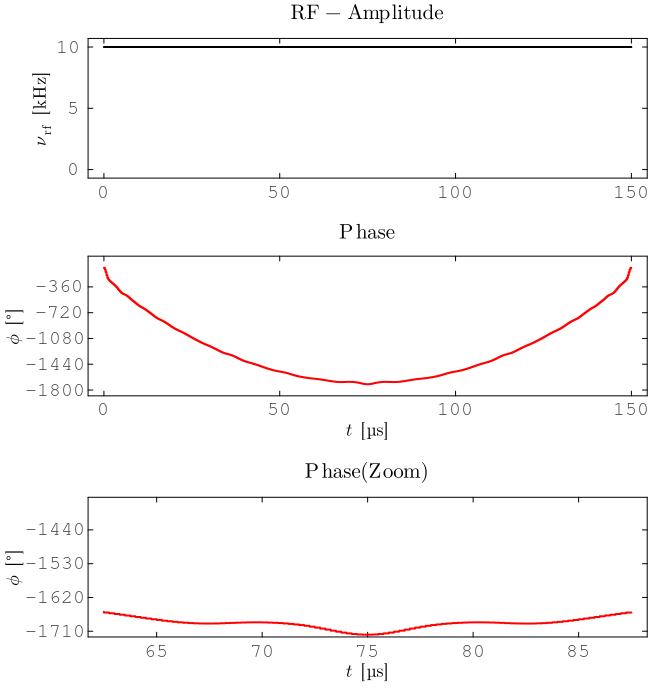

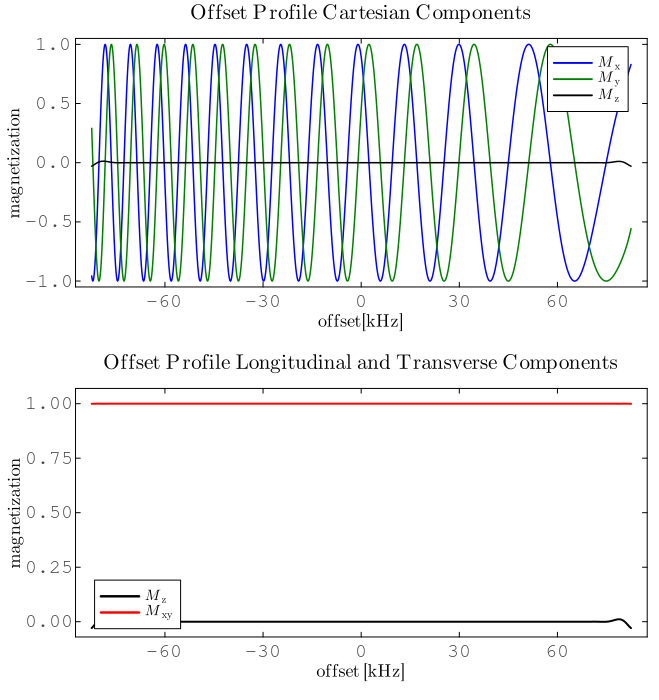
**

**ΔΩ/ν_rf_ = 20**

**
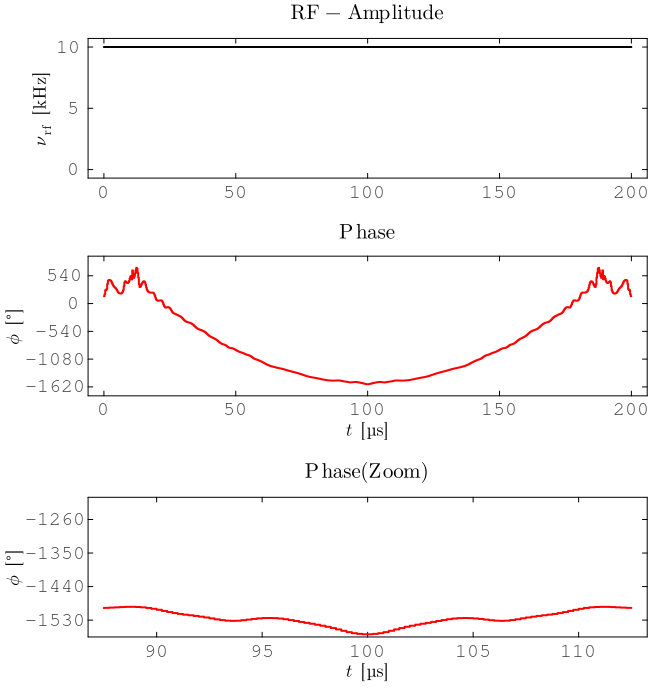

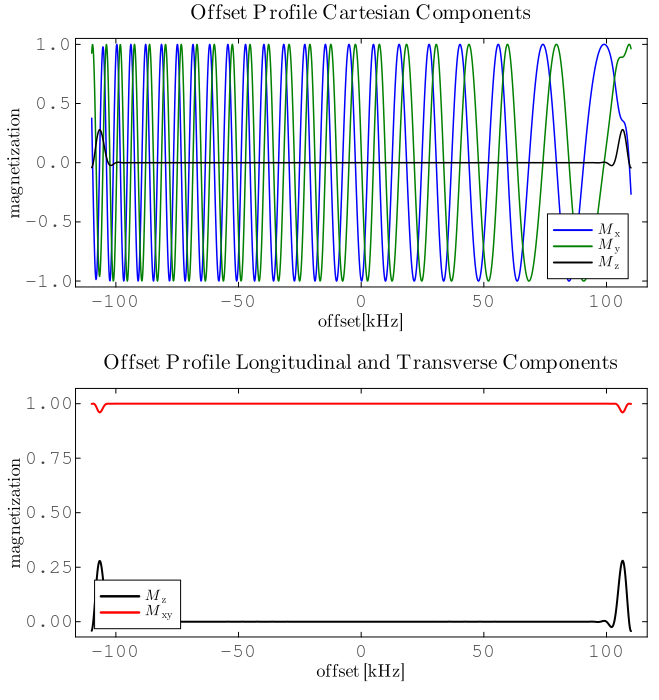
**

**ΔΩ/ν_rf_ = 25**

**
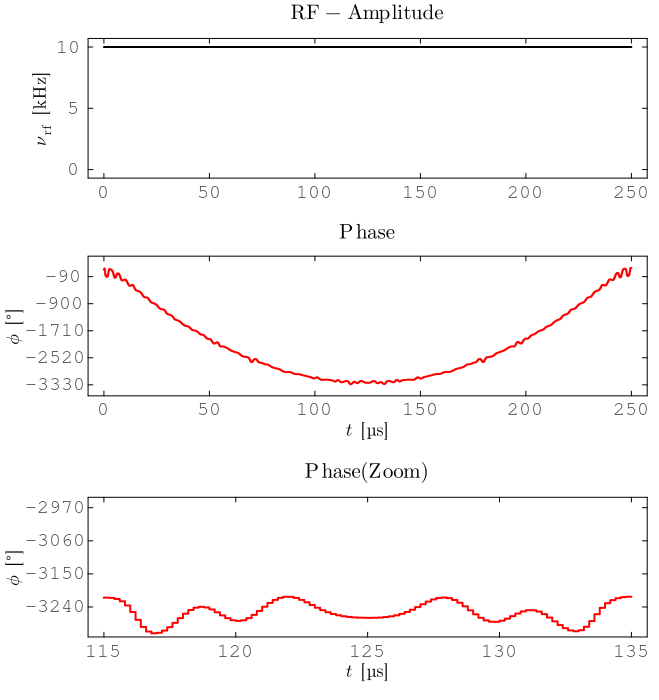

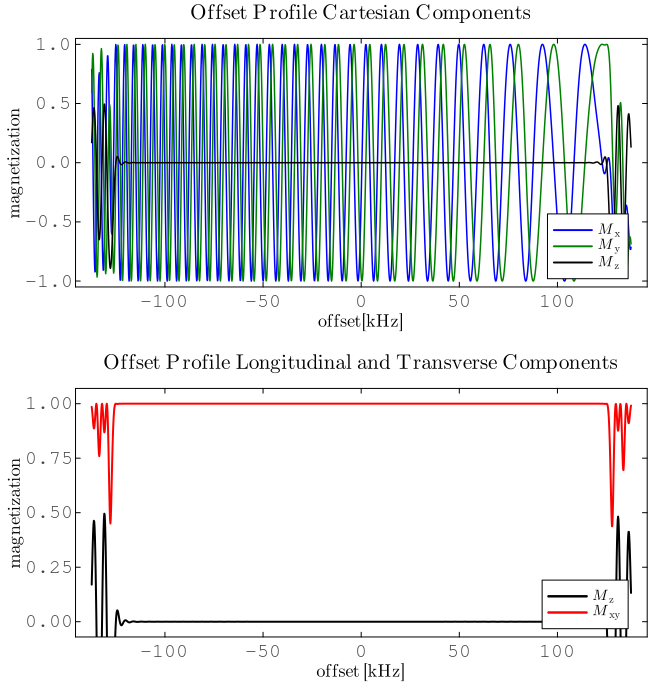
**

**ΔΩ/ν_rf_ = 30**

**
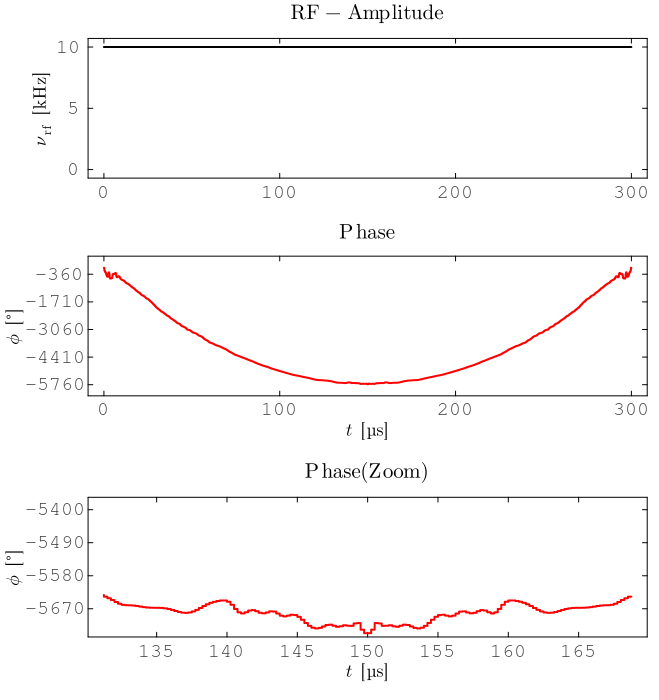

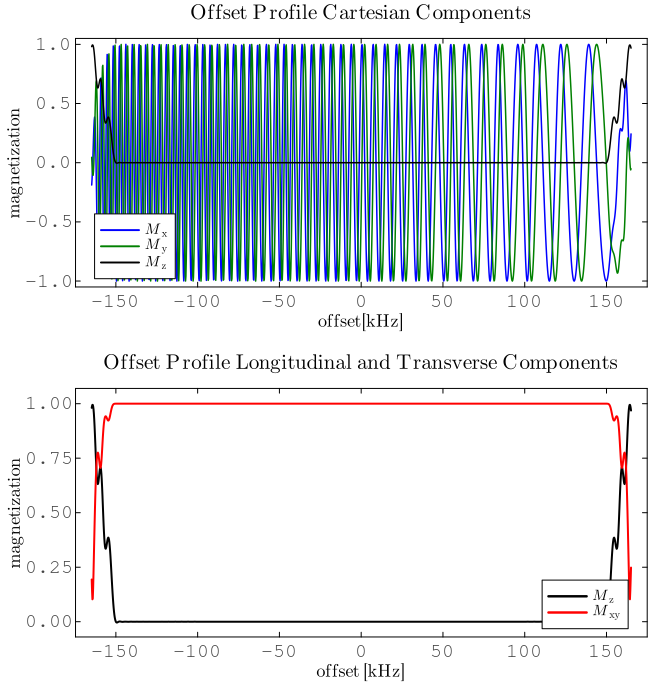
**

**ΔΩ/ν_rf_ = 40**

**
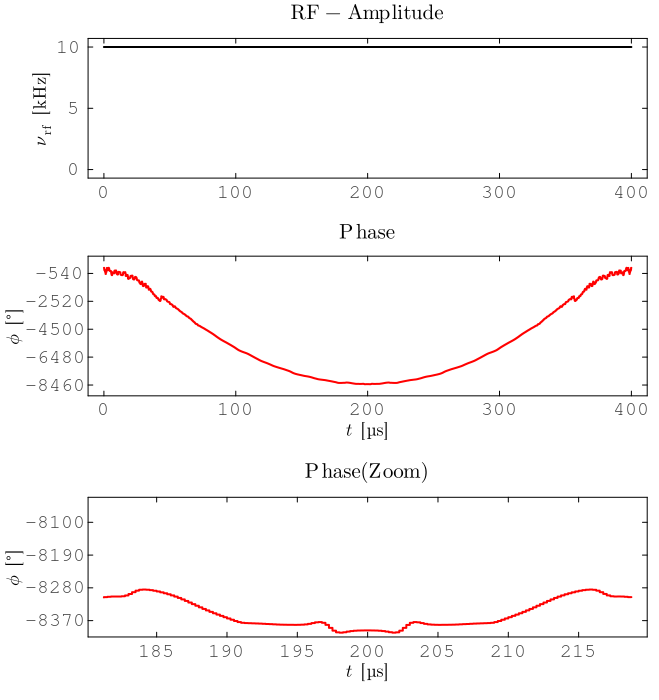

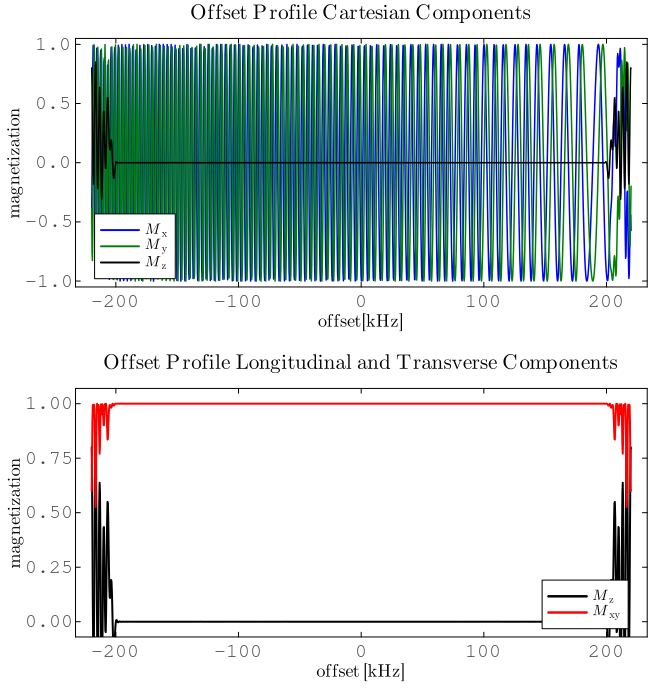
**

**ΔΩ/ν_rf_ = 50**

**
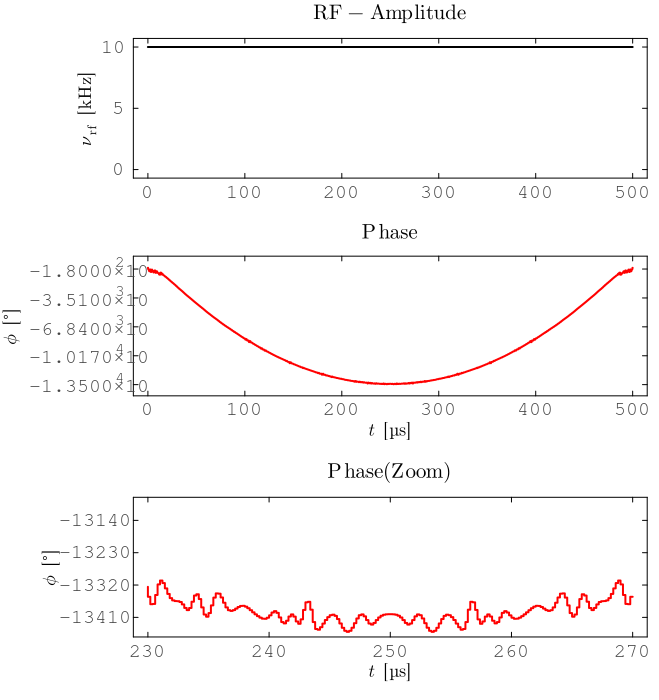

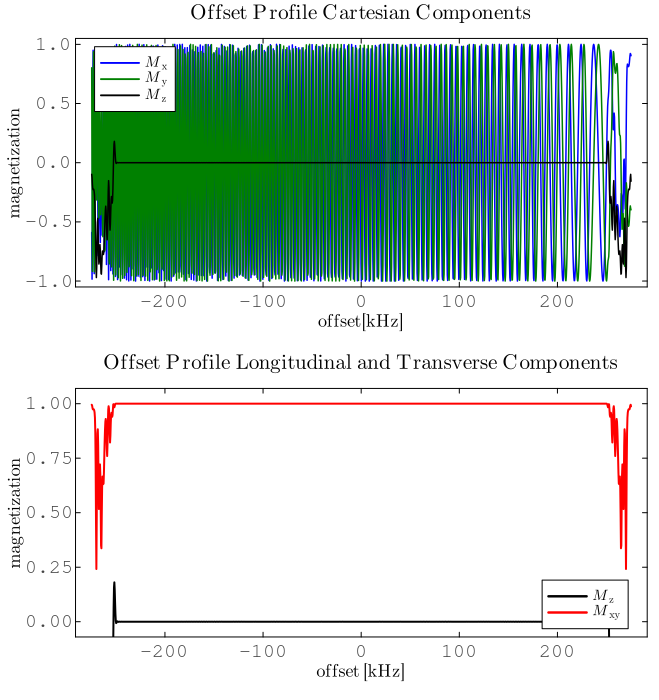
**

**ΔΩ/ν_rf_ = 75**

**
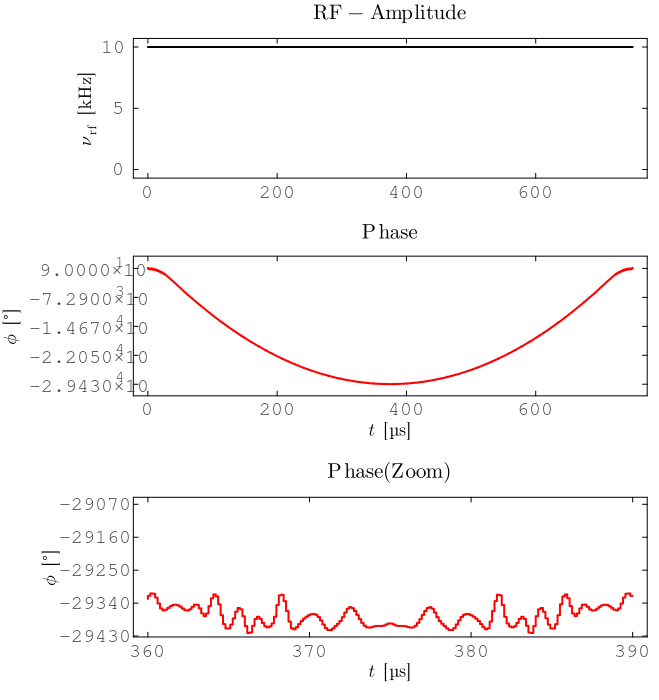

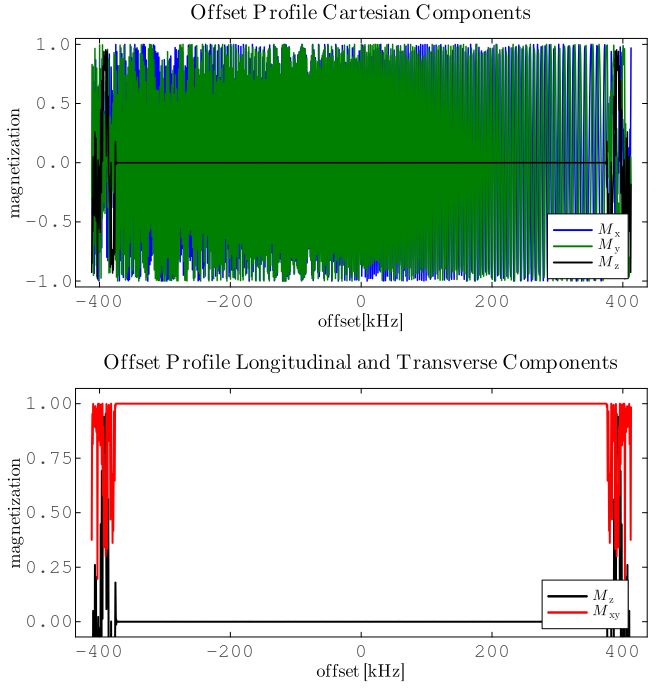
**

**ΔΩ/ν_rf_ = 100**


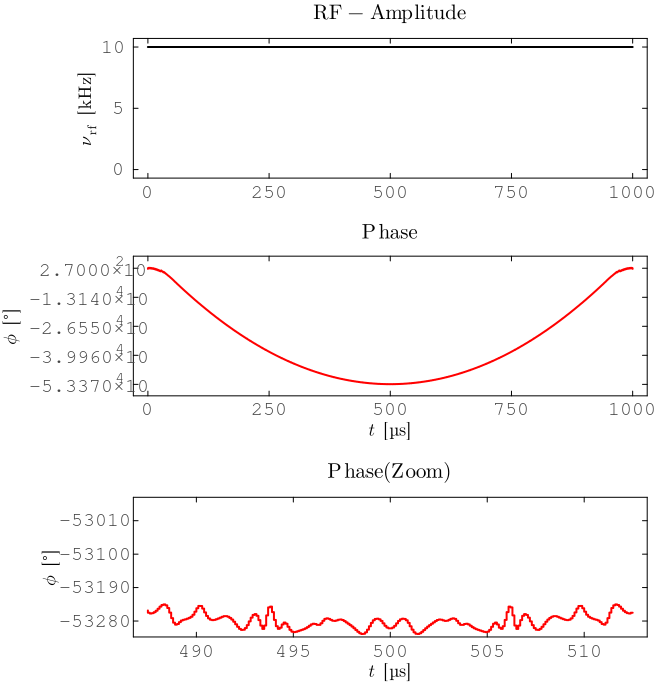

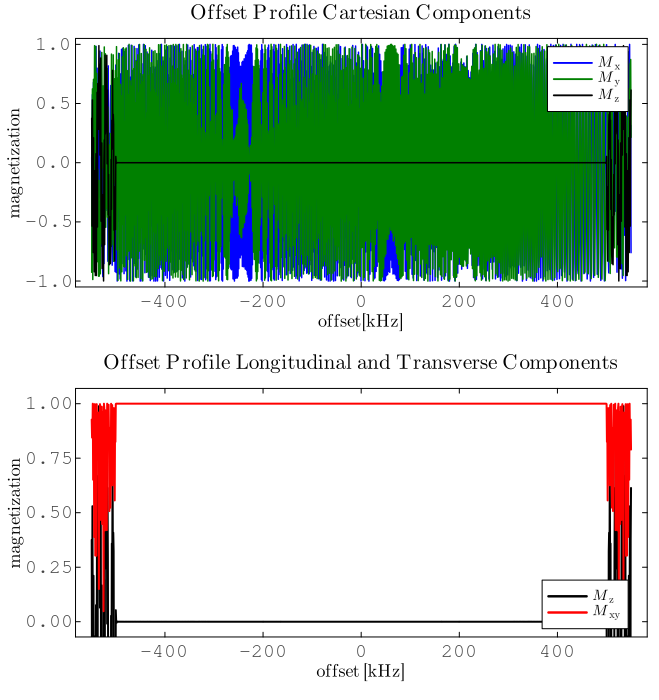


**ΔΩ/ν_rf_ = 150**

**
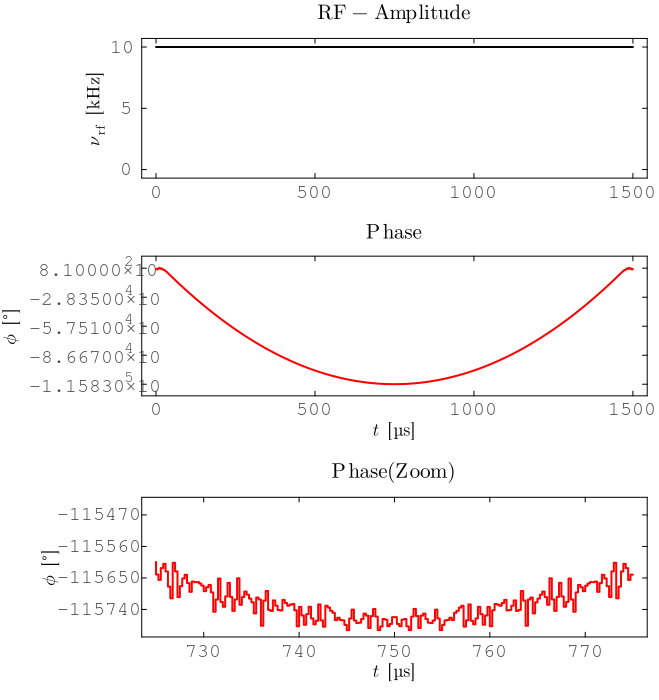

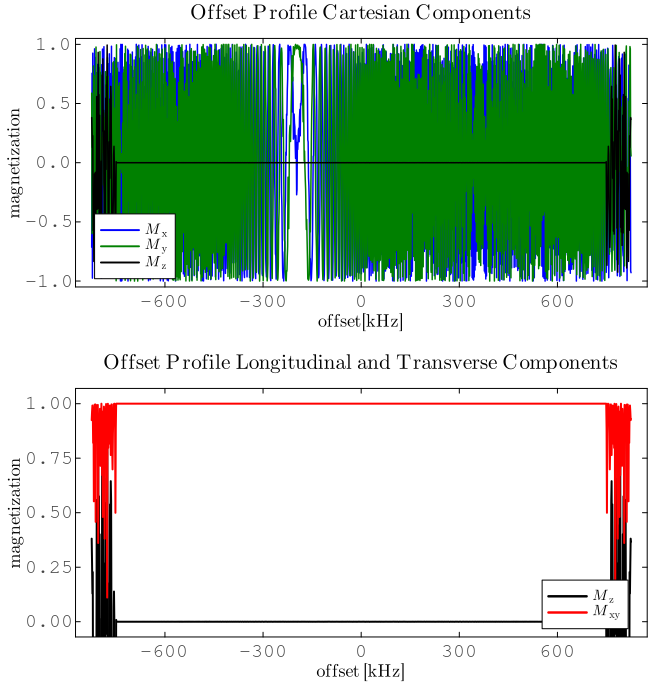
**

**ΔΩ/ν_rf_ = 200**

**
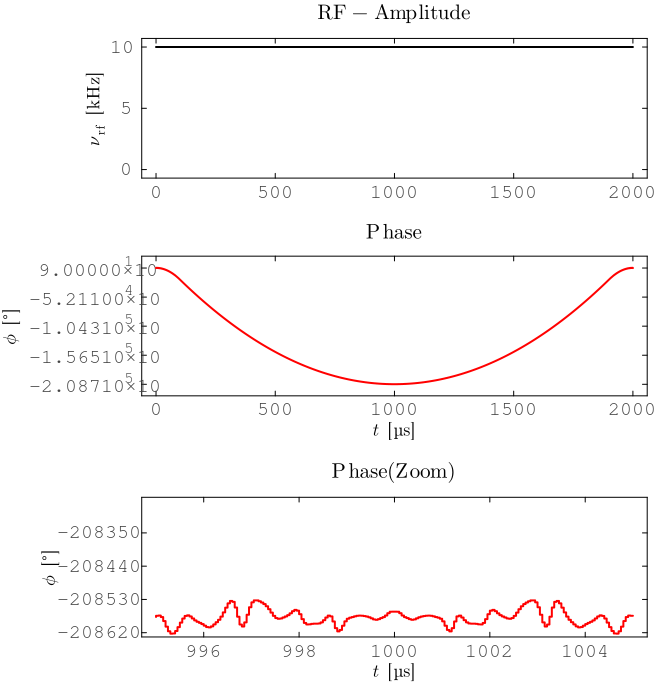

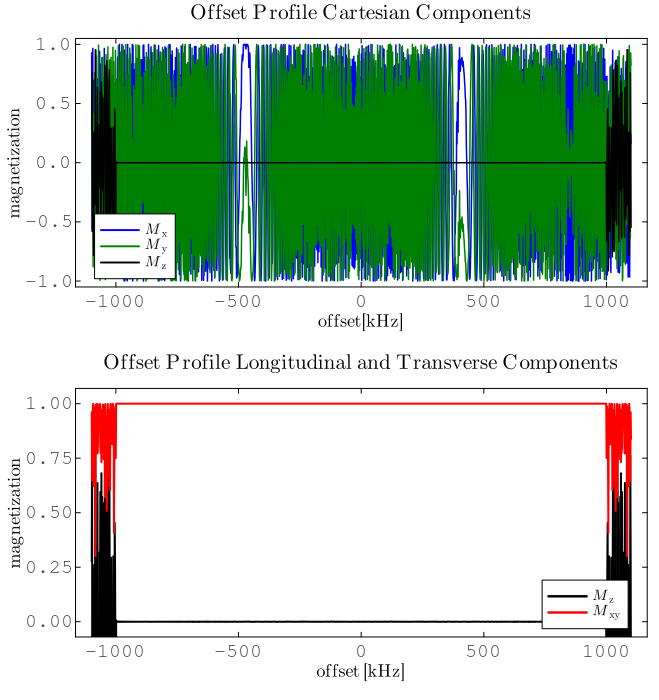
**

**ΔΩ/ν_rf_ = 300**

**
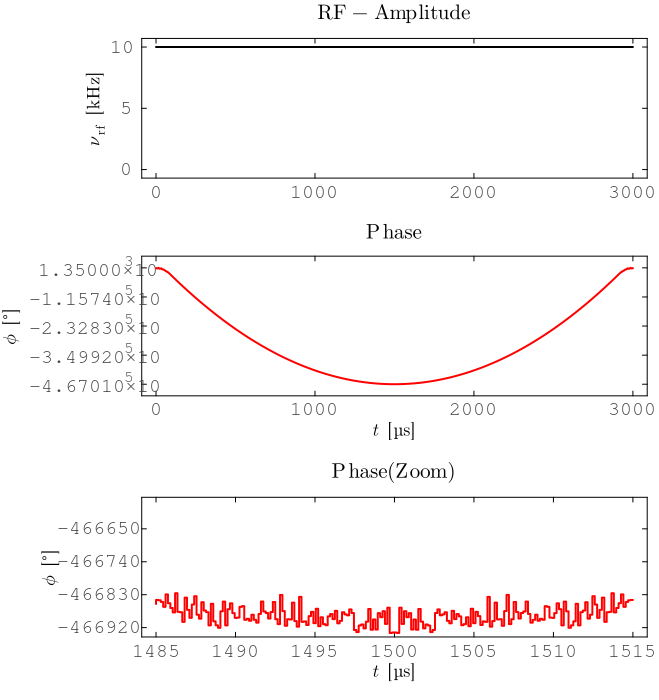

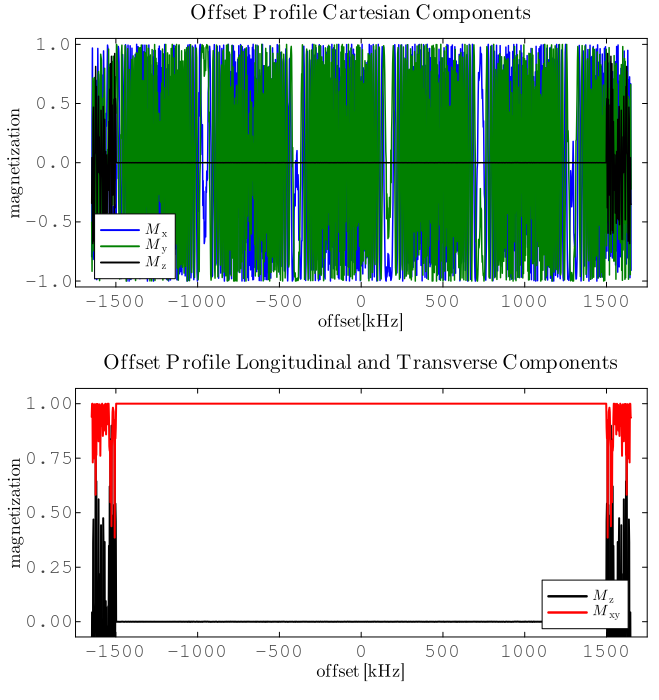
**

**ΔΩ/ν_rf_ = 400**

**
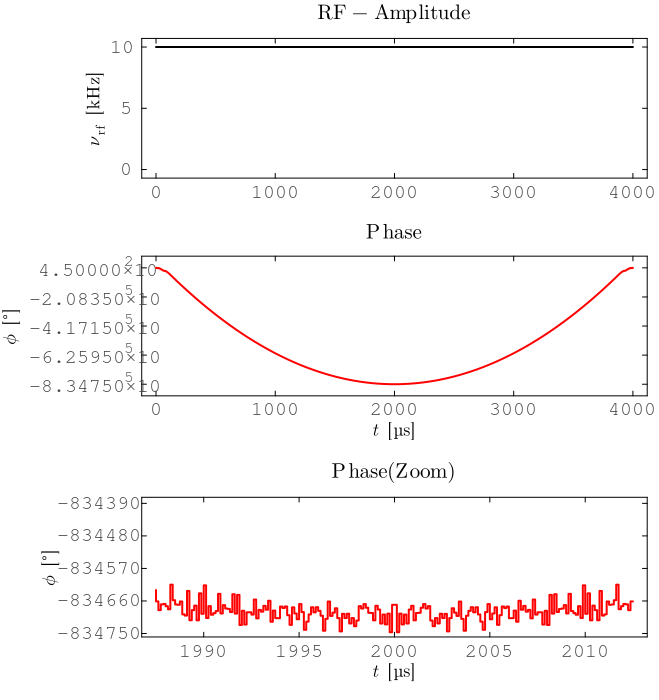

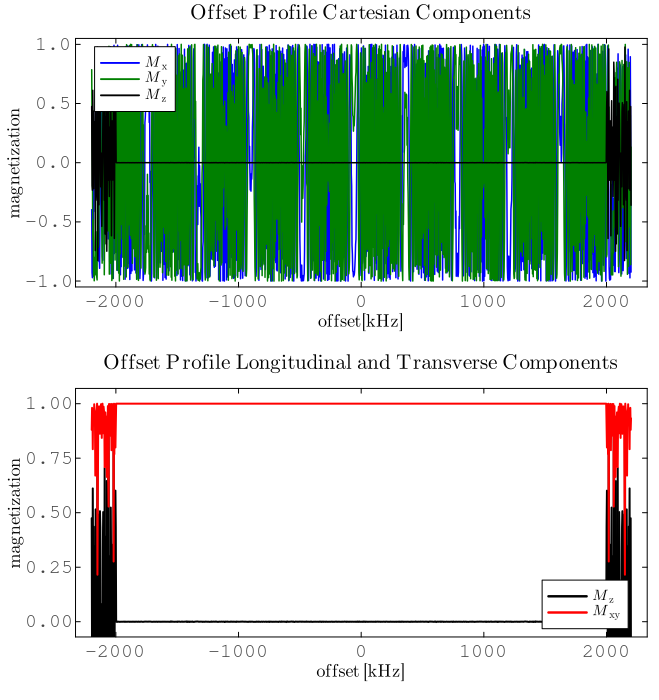
**

**ΔΩ/ν_rf_ = 500**

**
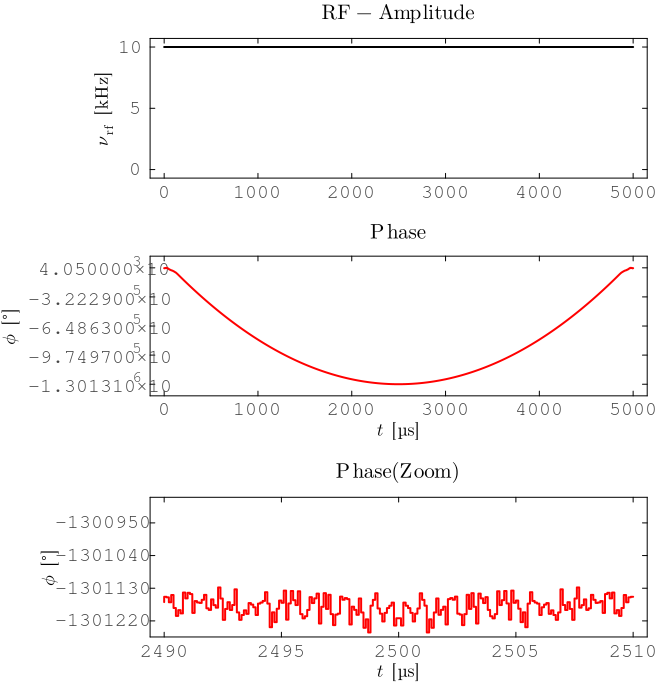

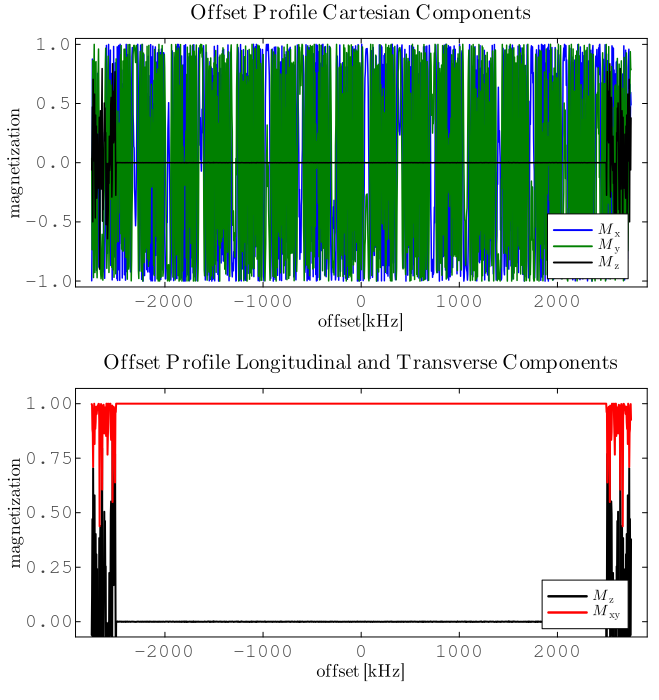
**

**ΔΩ/ν_rf_ = 600**


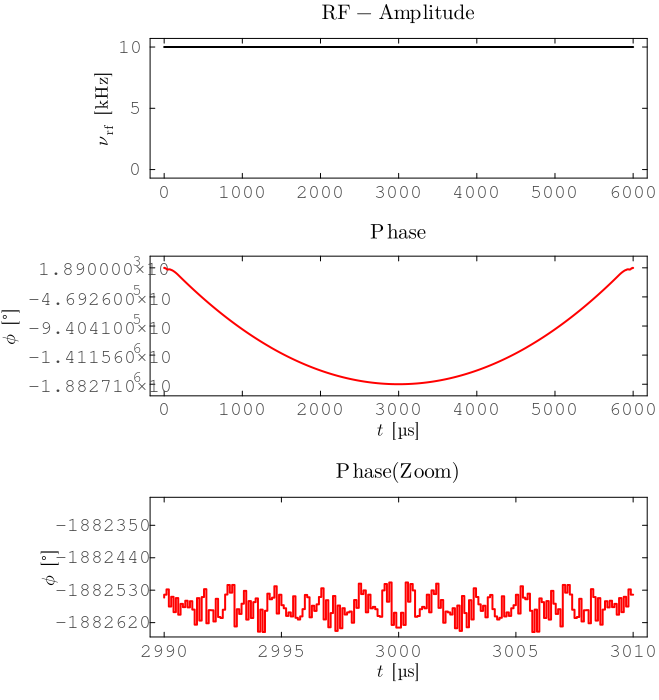

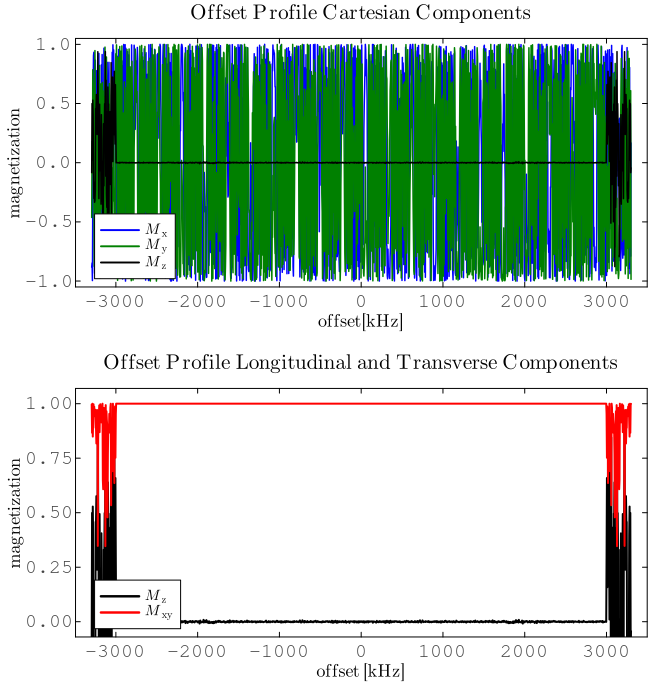


**B_1_-dependence of offset profile for the pulse used in the multinuclear experiment**


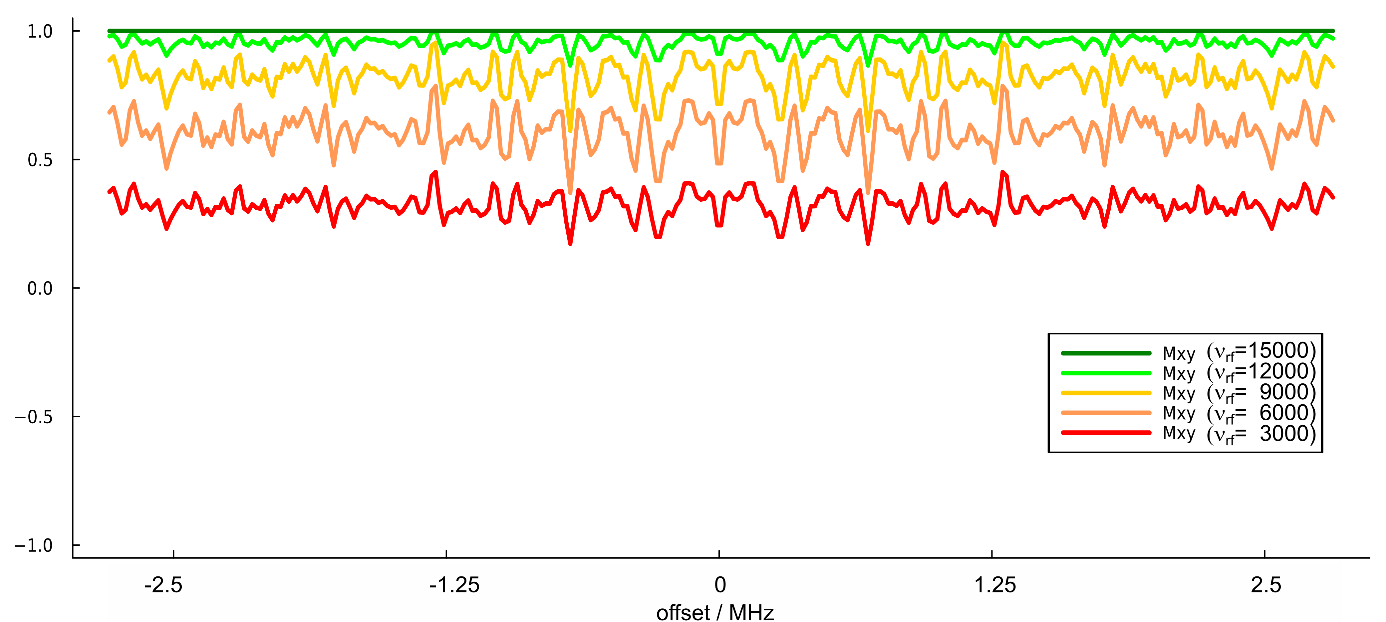


Offset profiles for different rf-amplitudes of the pulse shape optimized for a bandwidth-to-rf-amplitude-ratio of 400 and a pulse duration of 2.666 ms. Although significant local deviations are seen for rf-amplitudes deviating from the nominal 15 kHz, a rough sin(π ν_rf_/(2*15000 Hz)) dependence of the excited magnetization can be deduced.

**Influence of probe Q for extreme bandwidths/offsets**


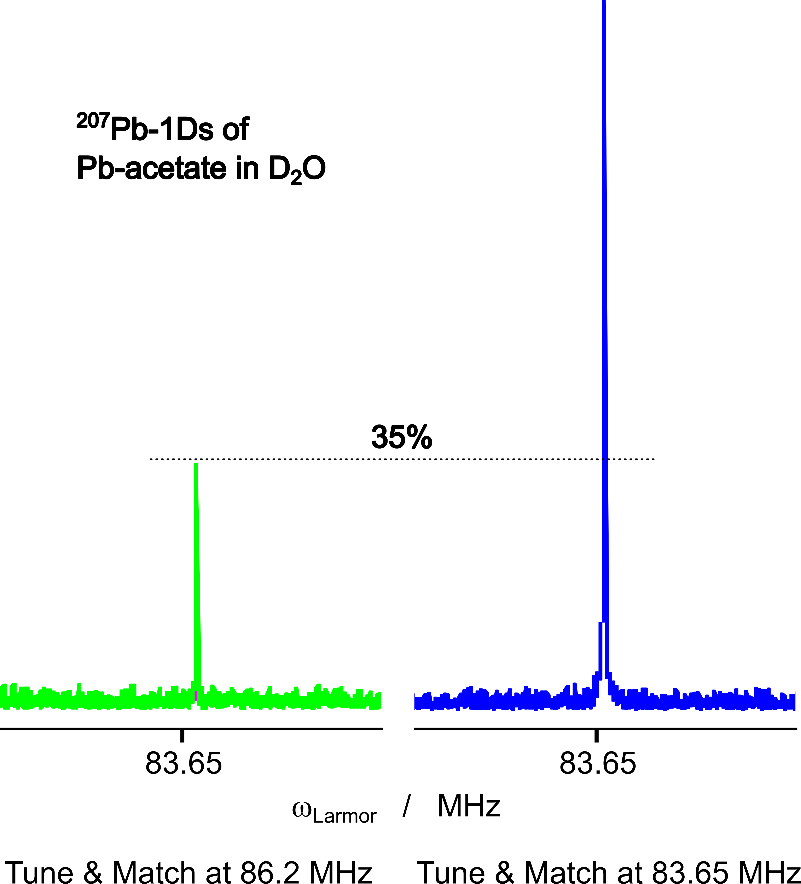


Spectra for Pb-acetate were recorded with tuning and matching performed for the on-resonant Larmor frequency of approximately 83.65 MHz and tuning and matching for the center of the multinuclear spectrum of Fig. 4 of the main text (~86.2 MHz). All other parameters were kept identical. Using the rough formula deduced in the previous section, an effective rf-amplitude at an offset of 2.55 MHz can be estimated to approximately 3500 Hz, i.e. ~24% of the rf-amplitude of 15000 kHz obtained with tuning and matching on-resonance.

The reduction of the rf-amplitude with offset is mainly influenced by the Q of the probe and the probe design in general. Probes with a broadband design usually come with reduced sensitivity, but would not have the issue of reduced rf-amplitudes at spectral edges. Standard probes, like the one used in this study, allow uncompromised performance within a frequency range of approximately ±500 kHz.

The overall performance for an extreme bandwidth like the 6 MHz of Fig. 4 could, in principle, be improved by increasing the nominal rf-amplitude and thereby decreasing the on-resonant performance, but increasing the overall performance over the entire spectral width. We did not try this here. The spectral width of 6 MHz might also be obtained in a series of 6 spectra with 1 MHz bandwidth, which would result in slightly higher S/N for the extreme offsets using the standard probe and nominal rf-amplitude for the same overall measurement time, but significantly decreased sensitivity in the central region. Using the increased nominal rf-amplitude, the single spectrum approach should outperform the 6 sequential spectra over the entire bandwidth.

The offset-dependent rf-amplitude due to the probe Q can in principle be included in pulse optimizations, leading generally to increased pulse lengths, but improved excitation performance. It requires, however, the detailed knowledge of the offset-dependence of a specific spectrometer/probe combination prior to optimization and therefore poses a limit to generality, as a different setup requires a different optimized pulse shape.
